# High-Throughput Microelectrode Arrays for Precise Functional Localization of the Globus Pallidus Internus

**DOI:** 10.34133/cbsystems.0123

**Published:** 2024-05-23

**Authors:** Yuxin Zhu, Luyi Jing, Ruilin Hu, Fan Mo, Qianli Jia, Gucheng Yang, Zhaojie Xu, Meiqi Han, Mixia Wang, Xinxia Cai, Jinping Luo

**Affiliations:** ^1^ State Key Laboratory of Transducer Technology, Aerospace Information Research Institute, Chinese Academy of Sciences, Beijing 100190, China.; ^2^ School of Electronics, Electrical and Communication Engineering, University of Chinese Academy of Sciences, Beijing 100049, China.

## Abstract

The globus pallidus internus (GPi) was considered a common target for stimulation in Parkinson’s disease (PD). Located deep in the brain and of small size, pinpointing it during surgery is challenging. Multi-channel microelectrode arrays (MEAs) can provide micrometer-level precision functional localization, which can maximize the surgical outcome. In this paper, a 64-channel MEA modified by platinum nanoparticles with a detection site impedance of 61.1 kΩ was designed and prepared, and multiple channels could be synchronized to cover the target brain region and its neighboring regions so that the GPi could be identified quickly and accurately. The results of the implant trajectory indicate that, compared to the control side, there is a reduction in local field potential (LFP) power in multiple subregions of the upper central thalamus on the PD-induced side, while the remaining brain regions exhibit an increasing trend. When the MEA tip was positioned at 8,700 μm deep in the brain, the various characterizations of the spike signals, combined with the electrophysiological characteristics of the β-segmental oscillations in PD, enabled MEAs to localize the GPi at the single-cell level. More precise localization could be achieved by utilizing the distinct characteristics of the internal capsule (ic), the thalamic reticular nucleus (Rt), and the peduncular part of the lateral hypothalamus (PLH) brain regions, as well as the relative positions of these brain structures. The MEAs designed in this study provide a new detection method and tool for functional localization of PD targets and PD pathogenesis at the cellular level.

## Introduction

PD is a common neurological disorder characterized by symptoms such as tremors, bradykinesia, and muscle rigidity. As the condition progresses, the quality of life of patients can be severely impacted. To address this issue, deep brain stimulation (DBS) has emerged as a novel treatment option for PD patients [1,2]. The globus pallidus internus (GPi), when used as a common target for DBS, has been shown to improve motor function and reduce symptoms like tremors and stiffness when stimulated with appropriate electrical impulses [3,4].

The localization of GPi presents many challenges. First, due to its deep location in the brain, the GPi is relatively concealed, increasing the difficulty of accurate positioning during surgery [5]. Additionally, the natural elasticity and fluidity of brain tissue cause inevitable tissue compression and displacement during DBS electrode implantation, resulting in unpredictable shifts in the previously determined GPi location and boundaries during surgery [6]. Conventional medical imaging localization techniques, such as magnetic resonance imaging or computed tomography, struggle when faced with this dynamic brain tissue. While these techniques can provide images of brain tissue in a static state [7], they cannot accurately reflect the real-time displacement of brain tissue during surgery.

Electrophysiological recording can distinguish features of neural signals [8], and single-channel microelectrode is commonly used for localization during surgery. However, the limitations of single-channel microelectrode in providing neural information are also a substantial issue. Due to their limitation of recording neural signals from a single direction, they struggle to provide a comprehensive and accurate representation of the GPi,s function and characteristics. This may lead to inaccurate identification of physiological features of the GPi by doctors, resulting in deviations during stimulation or even stimulation of incorrect areas. Even more problematic is that single-channel microelectrode can only respond to neural signals at individual locations. Therefore, they only produce localization effects when passing through brain region boundaries. Highly similar electrophysiological signals within the same brain region cannot aid in localization. Doctors may need to repeatedly insert electrode to find the optimal stimulation location. This repetitive process undoubtedly increases the duration of surgery and the risk to patients. It also potentially causes unnecessary damage to brain tissue. In this context, high-throughput microelectrode arrays (MEAs) have emerged as a crucial tool [9]. These arrays provide a robust platform for recording neural activity from multiple sites simultaneously, enabling precise localization of the GPi and its boundaries. This not only enhances the precision and safety of DBS surgeries but also opens up possibilities for individualized treatment plans. By accurately targeting the GPi, doctors can tailor stimulation parameters to each patient,s specific needs, leading to optimal therapeutic outcomes [10].

In the electrophysiological landscape of PD, β-band (13 to 35 Hz) oscillations are conspicuously present in several brain areas within the basal ganglia, notably the GPi. These β-band oscillations are tightly linked to the emergence of PD symptoms, especially akinesia and tremors, making them a pivotal biomarker for assessing disease severity. Exploiting these abnormal oscillations in the GPi of PD patients, MEAs can aid in achieving precise targeting. Moreover, MEAs equipped with high-throughput capabilities offer detailed neural insights at a fine spatial resolution, shedding light on the functional roles of the GPi.

This study introduces high-throughput MEAs that are fabricated utilizing microelectromechanical systems (MEMS) technology. The MEAs boast 64 recording sites strategically distributed across a depth of 3,160 μm, guaranteeing that electrodes traverse multiple brain regions during the implantation process. By analyzing neural activity patterns and characteristics across various locations, we can achieve precise localization of the GPi. This innovation paves the way for personalized DBS targeting in PD treatment. Additionally, the wealth of neural data obtained through MEA recordings can enhance our understanding of the underlying mechanisms and potential therapeutic strategies for neurological disorders like PD.

## Materials and Methods

### Design of positioning MEA

**Fig. 1.**
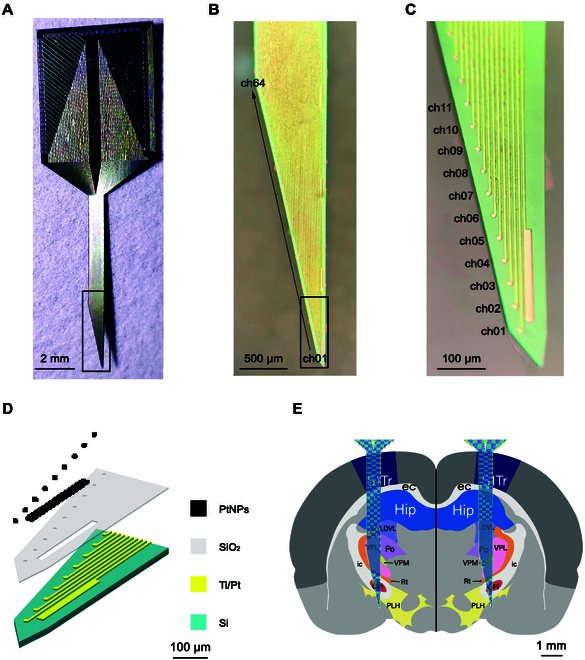
High-throughput MEAs and structures for electrophysiological localization of deep brain regions in GPi. (A) Photos of the complete MEAs in the flesh. (B) Microscopy of all 64 channels of a physical MEA tip. (C) Microscopic demonstration of multiple sites of the actual MEAs. (D) Layered presentation of the different material layers at the tip of the MEAs. (E) Brain mapping of the rat implanted in the surgical coronal plane.

In our study, we have designed and crafted a 64-channel single-shaft MEA. As depicted in Fig. 1A to C, each electrode site is a precisely circular 10-μm diameter, while the reference site is meticulously designed as a rectangular 200 μm × 15 μm feature. The spacing between these two detection sites is precisely maintained at 10 μm horizontally and 50 μm vertically. The 64-electrode sites are arranged in a diagonal pattern across a rectangular footprint spanning 3,160 μm in length and 640 μm in width. As shown in Fig. 1D, this electrode is tailored to reach a specific depth within the brain, precisely targeting the medial globus pallidus, which targets GPi with precision at a depth of 7,800 μm. With its total length of 9,000 μm, MEAs are capable of traversing the internal capsule (ic) surrounding the GPi and accessing distinct brain areas beneath it. During the predefined implantation trajectory, as MEAs come to a stop at any given position, the 64 sites do not necessarily all reside within the same brain region. This unique design enables us to capitalize on signal characteristics from at least two or more brain areas for comparison and localization of the boundaries between these regions. By leveraging this information, we can accurately determine the electrode’s position relative to the target and make timely adjustments to its orientation and coordinates.

This innovative design eliminates the need for repeated insertions within the brain, thereby enhancing the electrode’s positioning accuracy. Ultimately, this higher-precision localization paves the way for better treatment outcomes in DBS surgery for Parkinson’s disease (PD).

### Fabrication of MEAs

Silicon, with its excellent stability and mechanical strength, is an ideal material for serving as the substrate material for electrodes. On the one hand, it provides good mechanical strength to ensure the accuracy of positioning, and on the other hand, it allows the use of MEMS processes to perform various semiconductor processes on the silicon substrate to integrate more channel numbers. As shown in Fig. 1D, the processing of metal conductive layers, window layers, and outer layers is carried out on silicon-on-insulator (SOI) wafers [11]. First, a thermal oxide layer is deposited on the SOI to form the first layer of silicon dioxide. Then, a metal conductive layer pattern is lithographically transferred onto the SOI. After that, a Ti/Pt conductive metal layer is deposited. Next, another 800-nm-thick layer of silicon dioxide is deposited to form the surface insulation layer. The window pattern is used to etch and expose the detection sites and bonding pads. Finally, the outer shape of the electrode is obtained by using the outer layer pattern to deeply etch. By utilizing the reaction between KOH and Si, KOH is used to etch the SOI from the back, thereby releasing individual electrodes from the SOI [12].

In order to improve the detection performance of the electrode, platinum nanoparticles (PtNPs) were electrodeposited on the surface of the recording sites to reduce impedance and enhance the signal-to-noise ratio [13]. H_2_PtCl_6_ (48 mM) and Pb(CH_3_COO)_2_ (4.2 mM) were mixed in a 1:1 ratio to modify the detection site using PtNPs. MEA site as the working electrode and the platinum wire as the counter electrode were immersed in a prepared solution, and chronoamperometry (CA; −1.2 V, 30 s) on an electrochemical workstation (Gamry Instruments, USA) was used to achieve the electrodeposition of PtNPs.

### Modeling procedure of PD rats

Healthy adult male Sprague Dawley (SD) rats (Vital River, Beijing, China) weighing 250 to 300 g and displaying no abnormal rotation behavior will be selected for the study. The rats will be anesthetized using isoflurane, with the anesthesia concentration maintained at 1.5%. Subsequently, they will be fixed securely to the stereotaxic device (51650, Stoelting, USA).

Surgical operations were performed by cutting the scalp and exposing the skull, and the injection point coordinates were determined. The coordinates are the substantia nigra (SNc) (anterior posterior: −4.9 mm, medial lateral: 2.0 mm, dorsal ventral: −8.2 mm) and the medial forebrain bundle (MFB) (anterior posterior: −3.6 mm, medial lateral: 1.8 mm, dorsal ventral: −8.4 mm) of the right brain. The drug solution should be prepared by dissolving 6-OHDA (6-hydroxydopamine) in 0.2% ascorbic acid physiological saline to obtain a 2 μg/μl 6-OHDA solution. Four microliters of the drug solution is injected into each injection point, and then sutures for recovery. After the surgery, 0.1% apomorphine was injected subcutaneously into the neck of the rats once a week, aimed at inducing unidirectional rotation toward the control side. The rotation speed was then recorded. A mean speed greater than 7 r/min is considered as a successful unilateral PD rat model (Movie S1) [14]. Six SD rats were successfully used for signal measurements.

### Positioning and recording in the brain of PD rats

During the craniotomy, the coordinates on both sides (anterior posterior: −2.5 mm, medial lateral: ±2.9 mm) are exposed. In this study, a microthruster is used to drive the MEAs into the target coordinates at a speed of 1 μm/s.

During implantation, the tips of the MEAs are used to record signals at different depths with a step length of 50 μm. Each recording lasts for 10 min until the 9,000-μm single-shank MEA is fully implanted into the brain. The implantation path starts from the S1 somatosensory cortex (S1Tr) and passes through the external capsule (ec), hippocampus (Hip), dorsolateral thalamus (LDVL), posterior thalamic nuclear group (Po), ventral posteromedial nucleus of the thalamus (VPM), ventral posterolateral nucleus of the thalamus (VPL), reticular nucleus (Rt), ic, GPi, ic, and peduncular part of the lateral hypothalamus (PLH) (Fig. 1E). After the 64-electrode sites have reached a depth of 9,000 μm, they traverse the GPi, as well as the ic that surrounds it. Additionally, the electrode sites also examine brain regions located both above and below the ic. The aim of this traversing and examination is to achieve precise localization of the target brain region, as well as the identification of its boundaries and those of adjacent brain regions. A self-designed electrophysiological recording system [15] is used to record neural cell signals throughout the process. All the signal detection was performed at 37 °C to keep rats at a comfortable temperature.

The t-distribution expectation-maximization (t-EM) algorithm and V-search algorithm are used to extract the peak features through principal components analysis (PCA). The spike and local field potential (LFP) data are analyzed by Neuroexplorer. Additionally, all data in this paper are calculated as the mean ± SE.

## Results

### Surface modification of PtNPs

**Fig. 2.**
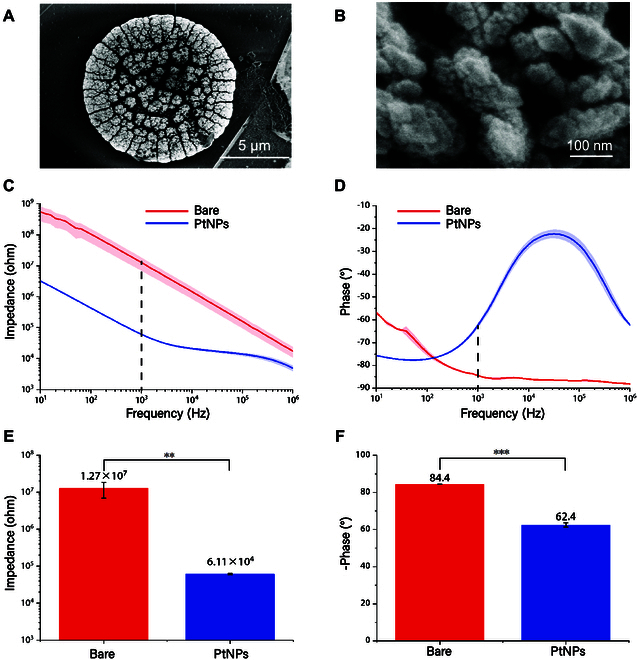
Modification of the site surface. (A) SEM images of sites modified by PtNPs. (B) Microsurface structure of PtNPs under SEM. (C) Impedance spectra of bare sites and sites modified with PtNPs. (D) Phase spectra of bare sites and sites modified by PtNPs. (E) Average impedance of bare and PtNP-modified microelectrode at 1 kHz. (F) Average phase of bare and PtNP-modified microelectrode at 1 kHz.

Figure 2A and B shows the scanning electron microscope (SEM) images of the sites after modification by PtNPs and the SEM images of the microscopic surface structure of PtNPs, and it can be observed that the PtNPs made the surface of the sites rough and porous and increased the surface area of the sites. In Fig. 2C and D, the impedance and phase spectra in the frequency range of 10 Hz to 1 MHz were obtained by scanning the bare electrode sites and the PtNP-modified electrode sites using electrochemical impedance spectroscopy (EIS). The average impedance of the electrode at 1-kHz frequency decreased from 12.7 ± 5.8 MΩ to 61.1 ± 2.3 kΩ, and the average phase increased from −84.40 ± 0.07° to −62.43 ± 1.16°.

Data are mean ± SE. One-way repeated-measures analysis of variance (ANOVA): *P < 0.05; **P < 0.01; ***P < 0.001.

### The recorded electrophysiological signals from multi-brain regions

**Fig. 3.**
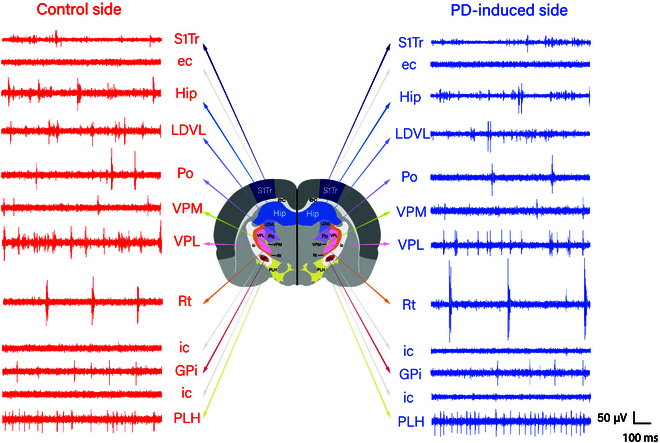
Raw electrophysiological signals with distinct brain region characteristics recorded in different brain regions passed on both sides of the implantation process.

Figure 3 shows the raw electrophysiological signals from the control side and PD-induced side of unilateral PD rats. During the implantation process of PD rats’ bilateral brain regions, multiple brain regions exhibit distinct patterns. It is evident that distinct neural signals emerge from various brain regions due to their diverse functions. During the actual testing, neural cells in various brain regions became less active due to anesthesia [5], [16–18]. To enhance understanding of the firing characteristics of these brain regions, we present the raw electrophysiological signals from channels with more prominent spike firings in distinct brain areas. In the implantation trajectory, signal regularity was generally low in the cortex and hippocampus, featuring multiple firing patterns. As MEAs progressed into the thalamus, the observation revealed that there were more cells with intense firing rates, along with a notable increase in firing rate regularity. Both tonic and clustered firing rates increased significantly.

### Characteristics of electrophysiological signals in brain regions during implantation

**Fig. 4.**
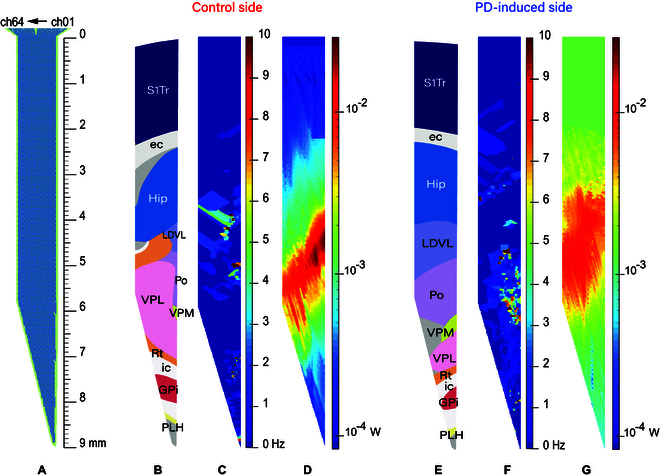
LFP power and spike firing rate planer thermograms of bilateral brain implantation trajectories. (A) Electrodes and their corresponding implantation depths. (B) Implantation trajectory mapping for the control side. (C) Spike firing rate thermogram of the control side. (D) LFP power thermogram of the control side. (E) Implantation trajectory mapping for the PD-induced side. (F) Spike firing rate thermogram of the PD-induced side. (G) LFP power thermogram of the PD-induced side.

Due to the electrodes, design and meticulous experiments, a comprehensive neural signal coverage was achieved along the entire implantation trajectory with a high density. In this study, we utilized a pixel size of 10 μm × 10 μm (reference site size) to construct a square array of 64 × 900. The LFP power and spike firing rate were visualized using color-coding to create a two-dimensional heatmap of the implantation trajectory. This approach allows for a comprehensive representation of the rich data obtained. Figure 4C and F represents the spike firing rate, Fig. 4D and G represents the LFP power, and Fig. 4B and E represents the implantation trajectory displayed on the brain atlas.

First, the coexistence of the control side and the PD-induced side is characterized. As shown in Fig. 4D and G, the heatmap reveals that the upper and middle regions of the thalamus in both hemispheres exhibit higher LFP power, whereas the LFP power in the S1Tr, Hip, and deep brain nuclei located beneath the thalamus is relatively lower compared to the thalamus. The LFP power in multiple subregions within the thalamus is generally on the order of tens of milliwatts, while the LFP power in other brain regions is on the order of several milliwatts or even lower. The trend of spike firing rate is basically consistent with that of LFP power, and there are often more cells with high firing rate in regions with higher LFP power.

Second, when comparing the heatmaps on both sides, it is observed that the field potential power in the S1Tr, Hip, and deep brain nuclei on PD-induced side significantly increases, while the upper and middle part of the thalamus shows a significant decrease. This is consistent with the neural circuit model of PD [19], where the lack of dopamine in the SNc of the PD-induced side leads to excessive GPi output, resulting in excessive inhibition in the thalamus and motor cortex.

Third, as clearly observable in the heatmaps, within the same brain region at similar locations, there are still nonuniform color blocks that vary with the shape of the electrodes. This is because during the implantation process, the brain tissue inevitably experiences compression and uneven rebound.

### Time domain analysis of neural signals from GPi and its peripheral regions to precise localization

**Fig. 5.**
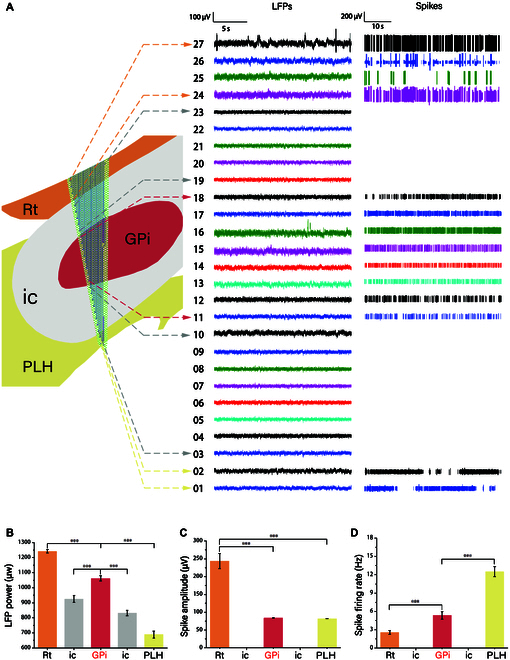
Localization analysis of Rt, supra-ic, GPi, lower-ic, and PLH on the PD-induced side. (A) Localization maps of electrode tip to GPi and electrode sites within brain regions of LFP and spike trains. (B to D) Histogram of LFP power, spike amplitude, and spike firing rate within each brain region.

When the tip of the MEAs is positioned near 8,700 μm, as shown in Fig. 5A, the oblique electrode point spans the GPi and its surrounding brain regions. Figure 5A shows the LFPs and spike trains of the loci within the five brain regions. From the raw data, Rt has a very characteristic discharge pattern. First, the high spike amplitude makes it localizable and distinguishable in the brain regions. Second, the firing rate interval is relatively consistent, indicating a more uniform distribution of spikes in the time domain. The average spike firing rate is 2.57 Hz. In addition, due to the high amplitude and consistency of spike firing rates within Rt, Rt had the highest average LFP power of 1,242 μW in the five brain regions. Downward into the ic, both raw data and LFPs became smooth, with a significant downward trend in the LFP power of about 25% and no longer showing an obvious spike signal. In the central region of the ic, the area where LFP power increased by approximately 10% and exhibited higher firing rates was identified as the GPi. The average firing rate of spikes in the GPi section was recorded at 5.35 Hz, with an average spike amplitude of 84 μV. After MEA tip penetrating out of the ic, the PLH brain region was reached, and the average power of the LFP decreased again compared with that of the ic, with a decrease of more than 10%, and the average amplitude of spike was about 82 μV, and the firing rate is dense but poor uniformity, from the spike trains, to 10 s as a cycle, every two to three cycles show dense firing rate after nearly a cycle of resting state, but the average firing rate is as high as 12.5 Hz. Using various spike-based features, GPi can be well localized.

### Frequency domain analysis of neural signals from GPi and peripheral regions to precise localization

**Fig. 6.**
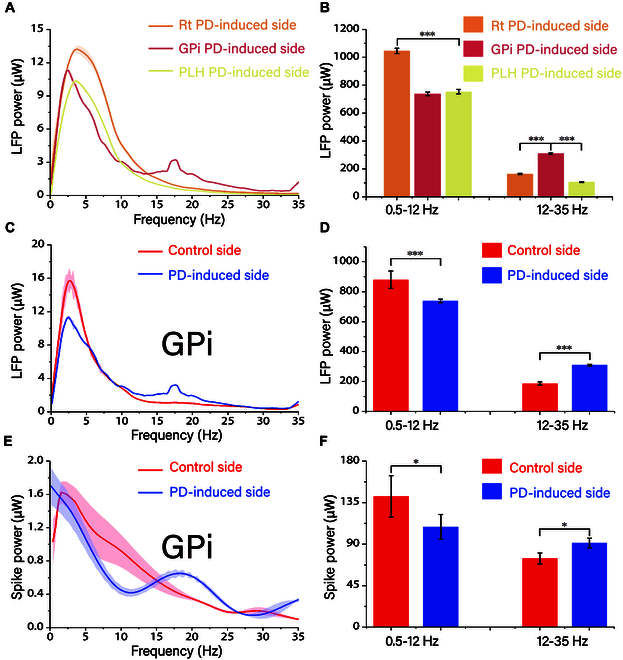
Characterization of brain regions using PSD maps. (A) Rt, GPi, and PLH superimposed LFP PSD maps of the PD-induced side. (B) Histogram analysis of LFP power in two bands for the three brain regions of the PD-induced side. (C) GPi LFP PSD maps of the superimposed maps of PD-induced side and control side. (D) Histogram analysis of the GPi LFP power in the two bands on both sides. (E) GPi spike PSD maps of the control side and PD-induced side and the superimposed maps on the both sides. (F) GPi spike power histogram in both bands on both sides.

Increased beta-band oscillations in the electrophysiologic profile of PD patients are thought to be a distinguishing feature of the lesion [20]. To better utilize this feature to help localization, we also individually analyzed Rt, GPi, and PLH on the PD-induced side and GPi on the PD-induced side using power spectral density (PSD) plots. The superimposed PSD maps of LFPs in the three brain regions on the PD-induced side are presented in Fig. 6A. The primary difference between the PSD maps of the PD-induced GPi and those of Rt and PLH is the emergence of a secondary bump within the frequency range of 12 to 35 Hz. This bump serves as a distinct lesion marker of PD, characterized by heightened β-segmental oscillations. This finding suggests that the analysis of short-term PSD maps can enhance our ability to discriminate and localize the affected GPi brain area.

Figure 6C displays the superimposed GPi LFP PSD of the control and PD-induced sides. The superposition maps show the decrease at 0.5 to 12 Hz and the increase at 12 to 35 Hz on the PD-induced side. Figure 6D shows the analysis of the histogram of the LFP power in two frequency bands, which decreased by 16% at 0.5 to 12 Hz and increased by about 66% at 12 to 35 Hz. This is in general agreement with the results of previous studies. In order to explore the microscopic causes of PD’s lesions at the cellular level, we also analyzed the spike signals of GPi brain regions using PSD, as shown in Fig. 6E and F, which showed a 23% decrease in 0.5 to 12 Hz and a 22% increase in 12 to 35 Hz using the same analysis as that of the PSD of LFPs. The results of spike analysis and LFP results are synchronized and relatively consistent.

## Discussion

In our work, the designed and prepared MEAs were shown to enable localization to multiple brain regions of the GPi and the implantation pathway. The sites were modified using PtNPs, which reduced the site impedance to 61.1 kΩ and improved the performance of the electrodes [21]. Compared with the four-channel localization electrodes mostly used in the market, the MEAs designed in this paper have a higher number of channels. Usually, the electrodes used for localization use ring-shaped sites with a diameter of 1.2 mm and a site width of 1.5 mm [22], which are more adequate for detecting a wider range of neural signals, but the electrode sites designed in this paper are able to achieve more accurate localization.

Taking into account the insights provided by Qian et al. [23], our study extends the understanding of neural signal analysis techniques by demonstrating that these techniques, customized for different segments of the implantation pathway, enable more precise localization. In Fig. 4, we obtain results that are consistent with Abe et al.’s [24] results for LFP power and suggest the use of LFP power as a key localization parameter throughout the implantation trajectory [25]. From Rt, into the small-sized nucleus parts of the deep brain, the importance of spike signals for localization is increased [26]. As shown by Li et al. [27], Rt has very high amplitude and strong features that are easy to distinguish. Upon entering the ic, spike signaling virtually disappears, whereas the rise in LFP power and regular issuance of spike signals measured at the posterior electrode site in the region of the ic near the center is evidence that the site detects GPi [28]. From stained brain sections [29], both the ic and GPi brain regions showed sparse and porous reticulation, poor tissue homogeneity, low cell density, and a relatively blurred border of the GPi. Taking into account the insights provided by Sil,s algorithm analysis methods in 2023 [30], our study extends the understanding of neural signal analysis techniques. When applying these techniques to PSD maps for analysis, we observed that the PSD of ic in the region near Rt and PLH exhibited a peak within the 0.5- to 12-Hz frequency range. Similarly, the PSD of the area close to GPi revealed two distinct peaks, one in the 0.5- to 12-Hz range and another in the 12- to 35-Hz range [31]. These findings demonstrate that the customized neural signal analysis techniques, when applied to different segments of the implantation pathway, enable more precise localization. Therefore, when locating the GPi, the PSD is first analyzed, and if there is a significant increase in the β segment, it indicates that the electrode is already in the vicinity of the GPi. Second, if there is a clear spike signal and the firing rate exceeds 3 Hz, the unit and position measured at that site is considered to have reached the nerve cells of the GPi. Finally, an increase in LFP power can be used to aid in validation. When considering the localization of GPi, it is advisable to utilize LFP for larger brain areas. For small-sized nuclei in a variety of brain localization atlases [32], electrode sites are smaller and more accurately localized using spike. Additionally, brain areas with distinctive features at specific angles due to diseases or their inherent characteristics can be better localized.

Furthermore, the site layout and size of high-throughput MEAs can be customized to meet individual needs at the cellular level. In the prospect of potential clinical applications, the length of the handle and the design of the rear interface can be appropriately adjusted. Compared to the currently available multi-channel ring electrodes on the market [22], MEAs offer a higher density of recording sites and layouts, enabling more precise single-cell level positioning [33]. This capability allows for accurate spatial mapping and localization, enabling the acquisition of a greater quantity of neural information during implantation. Through the analysis and discussion of extensive implantation data, MEAs can further provide support and assistance in elucidating the cellular-level pathogenesis of diseases [34].

### Conclusion

In this study, a novel implanted deep brain MEAs were designed and prepared for functional localization of GPi in PD. The MEAs have multiple recording sites that are scattered over the GPi and several brain areas in front of and behind it. Therefore, at a specific depth, the MEAs can synchronously localize multiple brain areas and their boundaries. This minimizes the damage caused by repeated insertion during localization surgery. The results showed that the MEAs can synchronously detect neural signals from the GPi and its upper and lower five brain areas. By using multiple features of spikes, the brain areas can be localized with the precision of individual neurons. Compared with the control side, the PSD analysis on the PD-induced side showed a decrease in power from 0.5 to 12 Hz and an increase in power from 12 to 35 Hz. This may be one of the microscopic reasons for the motor dysfunction in PD patients. These special oscillations also provide assistance for the localization of GPi. Because Parkinson,s DBS is one of the most important treatments for PD, precise targeting of the target site is critical to the effectiveness of the stimulation and avoiding damage to the surrounding brain areas. The MEA proposed in this article provides a reliable tool for locating deep brain micronuclei.

## Data Availability

All data needed to support the conclusions in the paper are provided in the paper and the Supplementary Materials.
