# Supplementary material for: High-Throughput Microelectrode Arrays for Precise Functional Localization of the Globus Pallidus Internus
Source: Cyborg Bionic Syst. 2024 May 23;5:0123. doi: 10.34133/cbsystems.0123 (PMC11112599; doi:10.34133/cbsystems.0123)
Supplement: Supplementary 1 — Fig. S1 Movie S1 [file cbsystems.0123.f1.zip › Supplementary Materials.docx]

Supplementary Materials

Fig. S1 DiL MEAs stained brain sections.

Supplementary video: Successful PD rat rotated towards the healthy side with an average speed greater than 7 r/min after APO injection.
