# Supplementary figures and images for: High-Throughput Microelectrode Arrays for Precise Functional Localization of the Globus Pallidus Internus
Source: Cyborg Bionic Syst. 2024 May 23;5:0123. doi: 10.34133/cbsystems.0123 (PMC11112599; doi:10.34133/cbsystems.0123)

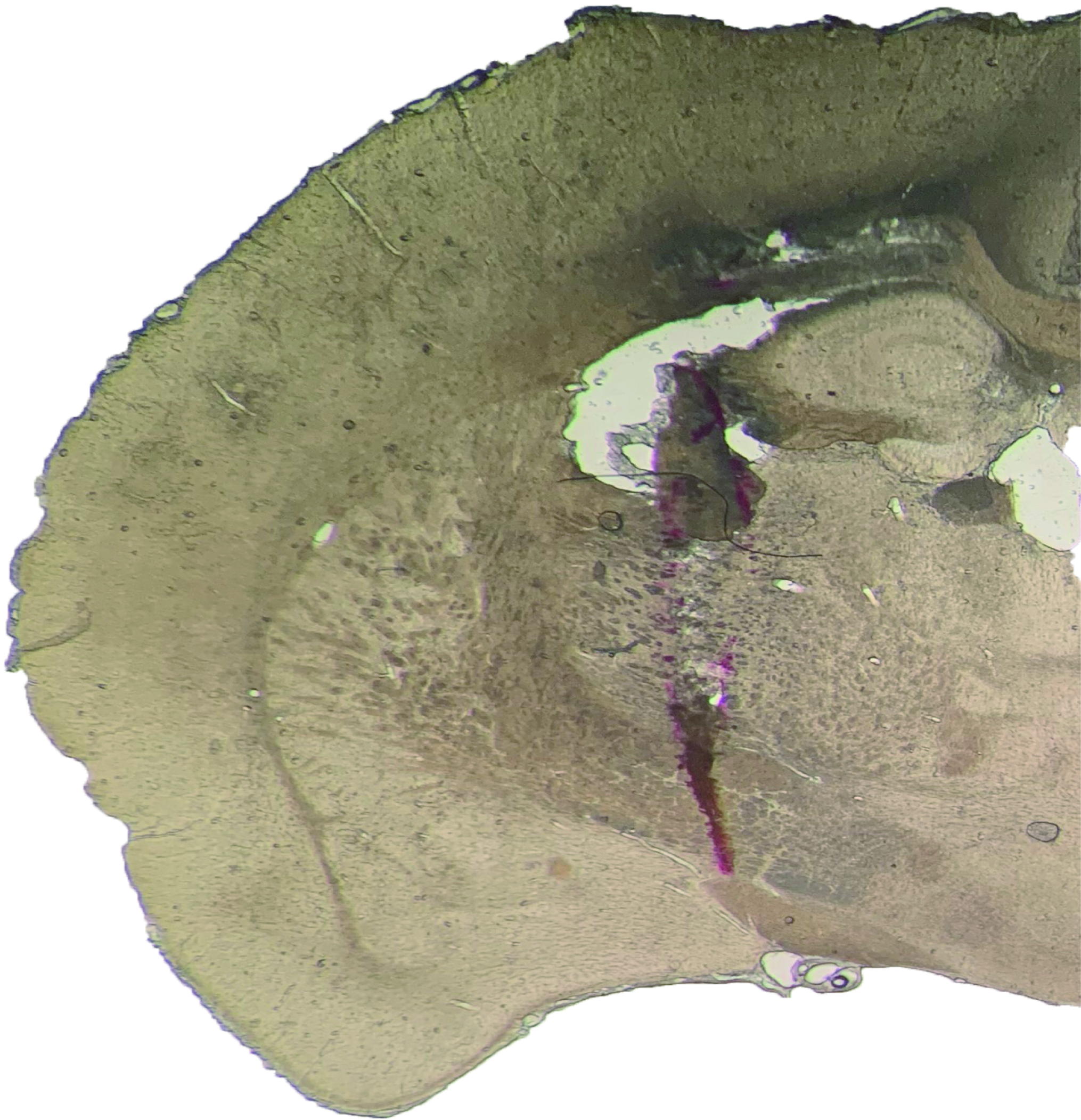

Supplement: Supplementary 1 — Fig. S1 Movie S1 [file cbsystems.0123.f1.zip › Fig. S1 DiL MEAs stained brain sections.tif]
